# A globally convergent QP-free algorithm for nonlinear semidefinite programming

**DOI:** 10.1186/s13660-017-1415-y

**Published:** 2017-06-23

**Authors:** Jian-Ling Li, Zhen-Ping Yang, Jin-Bao Jian

**Affiliations:** 1 College of Mathematics and Information Science, Guangxi University, Daxue Road 100, Nanning, Guangxi 530004 China; 2 School of Mathematics and Statistics, Guangxi Colleges and Universities Key Laboratory of Complex System Optimization and Big Data Processing, Yulin Normal University, Jiaoyu Road, Yulin, Guangxi 537000 China

**Keywords:** 90C30, 90C22, nonlinear semidefinite programmming, KKT conditions, QP-free algorithm, global convergence

## Abstract

In this paper, we present a QP-free algorithm for nonlinear semidefinite programming. At each iteration, the search direction is yielded by solving two systems of linear equations with the same coefficient matrix; $l_{1}$ penalty function is used as merit function for line search, the step size is determined by Armijo type inexact line search. The global convergence of the proposed algorithm is shown under suitable conditions. Preliminary numerical results are reported.

## Introduction

Consider the following nonlinear semidefinite programming (NLSDP for short):

1.1 $$\begin{aligned} \begin{aligned} &\min f(x) \\ &\quad \mbox{s.t. } \mathcal{A}(x)\preceq0; \\ &\hphantom{\quad \mbox{s.t.}}\ h_{j}(x)= 0, \quad j\in\mathcal{E}=\{1,2,\ldots,l \}, \end{aligned} \end{aligned}$$

where $f:R^{n}\rightarrow R$, $h_{j}\ (j\in{\mathcal {E}}): R^{n}\rightarrow R^{l}$ and $\mathcal{A}: R^{n}\rightarrow\mathcal{S}^{m}$ are continuously differentiable functions, not necessarily convex. $\mathcal{S}^{m}$ is a space whose elements are real symmetric matrices of size $m\times m$. ⪯ denotes the negative semidefinite order, that is, $A\preceq B$ if and only if $A-B$ is a negative semidefinite matrix.

NLSDP (1.1) has a broad range of applications such as eigenvalue problems, control problems, optimal structural design, truss design problems (see [1–3]). So it is desired to develop numerical methods for solving NLSDP (1.1).

In recent years, NLSDPs have been attracting a great deal of research attention [1, 3–25]. As is well known, NLSDP (1.1) is an extension of nonlinear programming, some efficient numerical methods for the latter are generalized to solve NLSDP. For example, Correa and Ramirez [26] proposed an algorithm which used the sequential linear SDP method. Fares *et al.* [27] applied the sequential linear SDP method to robust control problems. Freund *et al.* [4] also studied a sequential SDP method. Kanzow *et al.* [9] presented a successive linearization method with a trust region-type globalization strategy.

In addition, Kovara and Stingl [10] developed a computer code PENNON for solving NLSDP (1.1), where the augmented Lagrangian function method was used. Sun *et al.* [20] and Luo *et al.* [11, 22] proposed an augmented Lagrangian method for NLSDP (1.1), respectively. Sun *et al.* [19] analyzed the rate of local convergence of the augmented Lagrangian method for NLSDPs. Yamashita *et al.* recently proposed a primal-dual interior point method for NLSDP (1.1) (see [23]). The algorithm is globally convergent and locally superlinearly convergent under suitable conditions. Very recently Aroztegui [28] proposed a feasible direction interior point algorithm for NLSDP (1.1) with only semidefinite matrix constraint.

As we know, QP-free (also called SSLE) method is a kind of efficient methods for standard nonlinear programs (see [16]-[13]). In this paper, motivated from QP-free method for standard nonlinear programs, based on techniques of perturbation and penalty function, we propose a globally convergent QP-free algorithm for NLSDP (1.1). The construction of systems of linear equations (SLE for short) is a key point. Based on KKT conditions of NLSDP (1.1) and techniques of perturbation, we construct two SLEs skillfully. At each iteration, the search direction is yielded by solving two SLEs with the same coefficient matrix; An exact penalty function is used as the merit function for line search and the step size is determined by suitable inexact line search. The global convergence of the proposed algorithm is shown under some mild conditions.

The paper is organized as follows. In Section 2 we restate some definitions and results on NLSDP and matrix analysis. In Section 3 the algorithm is presented and its feasibility is discussed. The global convergence is analyzed in Section 4. Some preliminary numerical results are reported in Section 5 and some concluding remarks are given in the final section.

## Preliminaries

For the sake of convenience, some results on matrix analysis and NLSDP are restated in this section, which will be employed in the following analysis of the proposed algorithm. More introduction for theory of matrices should be seen in [21] and [6]. Denote by $R^{m\times n}$ the space of $m\times n$ real matrices, denote by $\mathcal{S}^{m}_{+}$ and $\mathcal {S}^{m}_{++}$ the sets of *m*-order symmetric positive semidefinite and positive definite matrices, respectively. The sets $\mathcal{S}^{m}_{-}$ and $\mathcal{S}^{m}_{--}$ are defined similarly.

### Definition 2.1

For any $A=(a_{ij}), B=(b_{ij})\in R^{m\times n}$, the inner product of *A* and *B* is defined by

2.1 $$ \langle A,B\rangle=\operatorname{Tr}\bigl(B^{\mathrm{T}}A \bigr)=\sum_{i=1}^{m}\sum _{j=1}^{n} a_{ij}b_{ij}, $$

where $\operatorname{Tr}(P)$ means the trace of the matrix *P*.

### Definition 2.2

[6]

For any $M\in R^{m\times m}$, let

2.2 $$ \operatorname{sym}(M)=\frac{1}{2}\bigl(M+M^{\mathrm{T}} \bigr), \qquad \operatorname{skw}(M)=\frac {1}{2}\bigl(M-M^{\mathrm{T}} \bigr), $$

$\operatorname{sym}(M)$ and $\operatorname{skw}(M)$ are called the symmetric part and the skew part of *M*, respectively.

Given a matrix $A\in\mathcal{S}^{m}$, let $\overline{m}=\frac {1}{2}m(m+1)$, define a map svec: $\mathcal{S}^{m}\rightarrow R^{\overline{m}}$:

$$ \operatorname{svec}(A)=(a_{11}, \sqrt{2}a_{21}, \ldots, \sqrt{2}a_{m1}, a_{22}, \sqrt{2}a_{32}, \ldots, \sqrt{2}a_{m2}, \ldots, a_{mm})^{\mathrm{T}}, $$

and the map $\operatorname{smat}:R^{\overline{m}}\rightarrow\mathcal{S}^{m}$ is defined to be the inverse of svec. Then the inner product of matrices is indicated by

2.3 $$ \langle A,B\rangle=\operatorname{svec}(A)^{\mathrm{T}} \operatorname{svec}(B), \quad \mbox{for } A, B\in\mathcal{S}^{m}. $$

### Definition 2.3

[21]

For any $A, B \in R^{m\times m}$, the symmetric Kronecker product, denoted by $A \otimes_{s} B$, is a mapping on a vector $u=\operatorname{svec}(U)$ where *U* is an $m\times m$ symmetric matrix and is defined as

2.4 $$ (A\otimes_{s} B)u=\frac{1}{2}\operatorname{svec} \bigl(BUA^{\mathrm{T}}+AUB^{\mathrm{T}}\bigr). $$

For any matrix $U\in\mathcal{S}^{m}$, it is verified that the following equality is true:

2.5 $$ (A\otimes_{s} B)\operatorname{svec}(U)= \operatorname{svec}\bigl(\operatorname{sym}(BUA)\bigr). $$

Note that the linear operator $A\otimes_{s} B$ is defined implicitly in (2.4). In Appendix of [21] a matrix representation of $A\otimes_{s} B $ is given as follows:

2.6 $$ A\otimes_{s}B=\frac{1}{2}Q(A\otimes B+B\otimes A)Q^{\mathrm{T}}, $$

where $A\otimes B=[a_{ij}B]$ ($i, j=1, 2, \ldots, m$) is the Kronecker product of *A* and *B*, *Q* is an orthogonal $\overline{m}\times m^{2}$ matrix (*i.e.* $QQ^{\mathrm{T}}=I_{\overline{m}}$), with the following property:

2.7 $$ Q\operatorname{vec}(U)=\operatorname{svec}(U),\qquad Q^{\mathrm{T}}\operatorname{svec}(U)=\operatorname{vec}(U), \quad \forall U \in\mathcal{S}^{m}, $$

where $\operatorname{vec}(U)=(u_{11},u_{21},\ldots,u_{m1},u_{12}, u_{22}, \ldots , u_{m2}, \ldots,u_{mm})^{\mathrm{T}}$.

### Remark 2.1

One choice for the matrix *Q* is given in the appendix of [21].

### Lemma 2.1

[21]

*For any* $A, B\in\mathcal{S}^{m}$, *the following results are true*:

1. $A\otimes_{s} B=B\otimes_{s} A$;
2. $(A\otimes_{s} B)^{\mathrm{T}}=A^{\mathrm{T}}\otimes_{s}B^{\mathrm{T}}$;
3. $(A\otimes_{s} B)(C\otimes_{s} D)=\frac{1}{2}(AC\otimes_{s} BD+AD\otimes_{s} BC)$;
4. *If* *A* *and* *B* *are symmetric positive definite*, *then* $A\otimes_{s} B$ *is positive definite*.

### Lemma 2.2

[28]

*If* $A, B\in S^{m}$, $A\succ0$ *and* $AB+BA\prec0$, *then* $B\prec0$.

### Lemma 2.3

*If* $A\in\mathcal{S}^{m}_{++}$, $B\in \mathcal{S}^{m}_{--}$, *then all eigenvalues of* *AB* *are less than zero*.

The proof is elementary and omitted here.

### Lemma 2.4

[28]

*If* $A\in\mathcal{S}^{m}_{++}$, $B\in\mathcal{S}^{m}_{-}$, *and they commute*, *then* $(A\otimes _{s}I_{m})^{-1}(B\otimes_{s}I_{m})\in\mathcal{S}_{-}^{\overline{m}}$.

### Lemma 2.5

*Suppose* $A\in\mathcal{S}^{m}_{++}$, $B\in\mathcal{S}_{--}^{m}$, *and they commute*, *then* $(A\otimes _{s}I_{m})^{-1}(B\otimes_{s} I_{m})\in\mathcal{S}_{--}^{\overline{m}}$.

### Proof

Since $A\in\mathcal{S}^{m}_{++}$, $B\in\mathcal{S}_{--}^{m}$, and they commute, there exists an orthogonal matrix $P\in R^{m\times m}$ such that

$$A=PD_{A}P^{-1}, \qquad B=P D_{B} P^{-1}, $$

where $D_{A}$ is a diagonal and positive definite matrix, and $D_{B}$ is a diagonal and negative definite matrix. It follows from Lemma 2.1(3) that

$$A\otimes_{s}I_{m}=\mathcal{T}\mathcal{D}_{A} \mathcal{T}^{-1}, \qquad B\otimes_{s}I_{m}= \mathcal{T}\mathcal{D}_{B}\mathcal{T}^{-1}, $$

where $\mathcal{T}=P\otimes_{s}P$, $\mathcal{D}_{A}=D_{A}\otimes_{s}I_{m}$ and $\mathcal{D}_{B}=D_{B}\otimes_{s}I_{m}$. We know from Lemma 2.1(2), (3) that $\mathcal{T}$ is orthogonal, from Lemma 2.1(4) that $\mathcal{D}_{A}$ is a diagonal and positive definite matrix, and $\mathcal{D}_{B}$ is a diagonal and negative definite matrix. Hence,

$$(A\otimes_{s}I_{m})^{-1}(B\otimes_{s}I_{m})= \mathcal{T}\mathcal{D}_{A}\mathcal{ D}_{B} \mathcal{T}^{-1}\in\mathcal{S}^{\overline{m}}_{--}. $$

 □

In the rest of this section we state the first order optimality conditions for NLSDP (1.1). For the sake of convenience, we first introduce some notations. Given a matrix valued function $\mathcal{A}(\cdot)$, we use the notation

$$D\mathcal{A}(x)= \biggl( \frac{\partial\mathcal{A}(x)}{\partial x_{1}},\ldots ,\frac{\partial\mathcal{A}(x)}{ \partial x_{n}} \biggr)^{\mathrm{T}} $$

for its differential operator evaluated at *x*, where $\frac{\partial \mathcal{A}(x)}{\partial x_{i}}$ denotes the partial derivative of $\mathcal{A}(x)$ with respect to $x_{i}$ with components $\frac{\partial a_{pq}(x)}{x_{i}}$ ($p,q=1,\ldots,m$). Then the derivative of $\mathcal {A}(\cdot)$ in the direction $d=(d_{1},\ldots, d_{n})^{\mathrm{T}}\in R^{n}$ at *x* denoted by $D\mathcal{A}(x)d$ is defined by

2.8 $$ D\mathcal{A}(x)d=\sum_{i=1}^{n}d_{i} \frac{\partial\mathcal {A}(x)}{\partial x_{i}}. $$

If we denote

2.9 $$ \nabla\mathcal{A}(x):= \biggl( \operatorname{svec}\biggl( \frac{\partial\mathcal{A}(x)}{\partial x_{1}}\biggr),\ldots,\operatorname{svec}\biggl(\frac{\partial\mathcal{A}(x)}{ \partial x_{n}} \biggr) \biggr)_{\overline{m}\times n}, $$

then by (2.8), the following equality is true:

2.10 $$ \operatorname{svec}\bigl(D\mathcal{A}(x)d\bigr)=\nabla \mathcal{A}(x)d. $$

The Lagrangian function of NLSDP (1.1) $L:R^{n}\times\mathcal{S}^{m} \times R^{l}\rightarrow R$ is defined by

2.11 $$ L(x,\Lambda,\mu)=f(x)+\bigl\langle \mathcal{A}(x),\Lambda\bigr\rangle +h(x)^{\mathrm{T}}\mu, $$

where $h(x)=(h_{1}(x), h_{2}(x), \ldots, h_{l}(x))^{\mathrm{T}}$. In view of (2.3), the above equality can be rewritten as follows:

$$ L(x,\lambda,\mu)=f(x)+\operatorname{svec}\bigl(\mathcal{A}(x) \bigr)^{\mathrm{T}}\lambda +h(x)^{\mathrm{T}}\mu, $$

where $\lambda: =\operatorname{svec}(\Lambda)$. The gradient of $L(x, \lambda, \mu)$ with respect to *x* is given as follows:

2.12 $$ \nabla_{x} L(x,\lambda,\mu)=\nabla f(x)+\nabla \mathcal{A}(x)^{\mathrm{T}}\lambda+\nabla h(x)\mu, $$

where $\nabla h(x)=(\nabla h_{1}(x), \nabla h_{2}(x), \ldots, \nabla h_{l}(x))$.

We are now in a position to restate the definition of the first order optimality conditions for NLSDP (1.1).

### Definition 2.4

[18]

For $x\in R^{n}$, if there exist a matrix $\Lambda\in\mathcal{S}^{m} $ and a vector *μ* ($\in R^{l}$) such that

2.13a $$\begin{aligned}& \nabla_{x}L(x,\Lambda,\mu)=0, \end{aligned}$$

2.13b $$\begin{aligned}& \Lambda\mathcal{A}(x)=0, \quad \Lambda\succeq0, \end{aligned}$$

2.13c $$\begin{aligned}& h(x)=0, \qquad \mathcal{A}(x)\preceq0, \end{aligned}$$

then *x* is called a KKT point of NLSDP (1.1).

### Remark 2.2

According to the Von Neumann-Theobald inequality, the complementarity condition $\Lambda{\mathcal {A}}(x)=0 $ has the following two useful equivalent forms:

$$\begin{aligned}& \operatorname{Tr}\bigl(\Lambda{\mathcal {A}}(x)\bigr)=0, \\& \lambda_{j}(\Lambda)\lambda_{j}\bigl({\mathcal {A}}(x) \bigr)=0,\quad \forall j\in\{ 1,2,\ldots,m\}. \end{aligned}$$

## The algorithm

In this section, we present our algorithm and show it is well defined. For the sake of simplicity, we introduce some notations:

$$\begin{aligned}& \Omega=\bigl\{ x\in R^{n}:\mathcal{A}(x)\preceq0, h(x)=0\bigr\} , \\& \mathcal{F}=\bigl\{ x\in R^{n}:\mathcal{A}(x)\preceq0\bigr\} , \qquad \mathcal{F}_{0}=\bigl\{ x\in R^{n}:\mathcal{A}(x)\prec0\bigr\} , \end{aligned}$$

that is, Ω is the feasible set of NLSDP (1.1).

In general, $\Lambda\mathcal{A}(x)$ is not guaranteed to be symmetric, so we consider $\operatorname{sym}(\Lambda\mathcal{A}(x))=0$ instead of $\Lambda \mathcal{A}(x)=0$. Then the three equalities of KKT condition (2.13a)-(2.13c) can be rewritten in the following form:

3.1 $$\begin{aligned} \begin{aligned} &\nabla f(x)+\nabla\mathcal{A}(x)^{\mathrm{T}} \lambda+\nabla h(x)\mu =0, \\ &\operatorname{svec}\bigl(\operatorname{sym}\bigl(\Lambda\mathcal{A}(x)\bigr) \bigr)=0, \\ &h(x)=0. \end{aligned} \end{aligned}$$

In order to solve (3.1) at each Newton iteration, we define a vector-value function $\varphi:R^{n+\overline{m}+l}\rightarrow R^{n+\overline{m}+l}$ as follows:

$$\varphi(x,\lambda,\mu)=\left ( \textstyle\begin{array}{@{}c@{}} \varphi_{{Lg}}(x,\lambda,\mu) \\ \varphi _{C}(x,\lambda,\mu) \\ \varphi_{h}(x,\lambda,\mu) \end{array}\displaystyle \right )= \left ( \textstyle\begin{array}{@{}c@{}} \nabla f(x)+\nabla\mathcal{A}(x)^{\mathrm{T}}\lambda +\nabla h(x)\mu \\ \operatorname{svec}(\operatorname{sym}(\Lambda\mathcal{A}(x))) \\ h(x) \end{array}\displaystyle \right ). $$

It follows from (2.5) and Lemma 2.1 that

$$\varphi_{{C}}(x,\lambda,\mu)=\operatorname{svec}\bigl( \operatorname{sym}\bigl(I\Lambda\mathcal {A}(x)\bigr)\bigr)=\bigl(I \otimes_{s}\mathcal{A}(x)\bigr)\operatorname{svec}(\Lambda)=(\Lambda \otimes _{s}I)\operatorname{svec}\bigl(\mathcal{A}(x)\bigr), $$

thus, the Jacobian of *φ* is

$$ \nabla\varphi(x,\lambda,\mu) =\left ( \textstyle\begin{array}{@{}c@{\quad}c@{\quad}c@{}} \nabla_{xx}^{2}L(x,\lambda,\mu) & \nabla\mathcal {A}(x)^{\mathrm{T}} & \nabla h(x) \\ (\Lambda\otimes_{s}I)\nabla\mathcal {A}(x) & I\otimes_{s}\mathcal{A}(x) & 0 \\ \nabla h(x)^{\mathrm{T}} & 0 & 0 \end{array}\displaystyle \right ). $$

Instead of the Hessian $\nabla_{xx}^{2} L(x,\lambda,\mu)$, we employ a positive definite matrix denoted by *H* which can be a quasi-Newton approximation or the identity matrix. A Newton-like iteration to solve (3.1) is given by the linear systems as follows:

3.2 $$\begin{aligned}& \left ( \textstyle\begin{array}{@{}c@{\quad}c@{\quad}c@{}} H & \nabla\mathcal{A}(x)^{\mathrm{T}} & \nabla h(x) \\ (\overline{\Lambda}\otimes_{s}I)\nabla\mathcal{A}(x) & I\otimes _{s}\mathcal{A}(x) & 0 \\ \nabla h(x)^{\mathrm{T}} & 0 & 0 \end{array}\displaystyle \right )\left ( \textstyle\begin{array}{@{}c@{}} x^{0}-x \\ \lambda^{0}-\overline{\lambda} \\ \mu^{0}-\mu \end{array}\displaystyle \right ) \\& \quad =-\left ( \textstyle\begin{array}{@{}c@{}} \nabla f(x)+\nabla\mathcal{A}(x)^{\mathrm{T}}\overline {\lambda}+\nabla h(x)\mu \\ \operatorname{svec}(\operatorname{sym}(\overline{\Lambda }\mathcal{A}(x))) \\ h(x) \end{array}\displaystyle \right ), \end{aligned}$$

where $(x,\overline{\Lambda},\mu)\in{\mathcal{F}}_{0}\times\mathcal {S}_{++}^{m}\times R^{l}$ is the current point, $(x^{0},\Lambda^{0},\mu^{0})\in \mathcal{F}\times\mathcal{S}_{++}^{m}\times R^{l}$ is the new estimates given by the Newton-like iteration, $\overline{\lambda}:=\operatorname{svec}(\overline{\Lambda})$ and $\lambda^{0}:=\operatorname{svec}(\Lambda^{0})$. Let $d^{0}=x-x^{0}$, we obtain from (3.2)

3.3a $$\begin{aligned}& Hd^{0}+\nabla\mathcal{A}(x)^{\mathrm{T}}\lambda^{0}+ \nabla h(x)\mu^{0}=-\nabla f(x), \end{aligned}$$

3.3b $$\begin{aligned}& (\overline{\Lambda}\otimes_{s}I)\nabla\mathcal{A}(x)d^{0}+ \bigl(I\otimes _{s}\mathcal{A}(x)\bigr)\lambda^{0}=0, \end{aligned}$$

3.3c $$\begin{aligned}& \nabla h(x)^{\mathrm{T}}d^{0}=-h(x). \end{aligned}$$

If $d^{0}=0$, then we have

$$\begin{aligned}& \nabla f(x)+\nabla\mathcal{A}(x)^{\mathrm{T}}\lambda^{0}+\nabla h(x)\mu ^{0}=0, \\& \bigl(I\otimes_{s}\mathcal{A}(x)\bigr)\lambda^{0}=0,\qquad h(x)=0. \end{aligned}$$

Since $\mathcal{A}(x)\prec0$, $I\otimes_{s}\mathcal{A}(x)$ is nonsingular and we have $\Lambda^{0}:=\operatorname{smat}(\lambda^{0})=0$, which implies that $\Lambda^{0}\mathcal{A}(x)=0$. Therefore, *x* is a KKT point. If $d^{0}\neq 0$, then $d^{0}$ is not guaranteed to be a feasible direction. To obtain a better search direction, we modify (3.3b) by introducing an appropriate right hand side, so we obtain another linear equations as follows:

3.4 $$\begin{aligned} \begin{aligned} &Hd^{1}+\nabla\mathcal{A}(x)^{\mathrm{T}} \lambda^{1}+\nabla h(x)\mu^{1}=-\nabla f(x), \\ &(\overline{\Lambda}\otimes_{s}I)\nabla\mathcal{A}(x)d^{1}+ \bigl(I\otimes _{s}\mathcal{A}(x)\bigr)\lambda^{1}=-\overline{ \lambda}\bigl\| d^{0}\bigr\| , \\ &\nabla h(x)^{\mathrm{T}}d^{1}=-h(x). \end{aligned} \end{aligned}$$

In order to ensure that SLEs (3.3a)-(3.3c) and (3.4) have a unique solution, respectively, the following assumption is required.

1. *For any* $x\in\mathcal{F}$, *the matrix*
  $$B(x)=\left ( \textstyle\begin{array}{@{}c@{\quad}c@{}} \nabla\mathcal{A}(x)^{\mathrm{T}} & \nabla h(x) \\ \mathcal{A}(x)\otimes_{s}I_{m} & 0 \end{array}\displaystyle \right ) $$
  *is full of column rank*.

The following lemma gives a sufficient condition of the assumption A1.

### Lemma 3.1

*For any* $x\in\mathcal{F}$, *if* $\mathcal{A}(x)\prec0$ *and* $\{\nabla h_{1}(x),\ldots,\nabla h_{l}(x)\}$ *is linearly independent*, *then* $B(x)$ *is full of column rank*, *i*.*e*., *the assumption* A1 *holds*.

### Lemma 3.2

*Let* *H* *be a positive definite matrix*. *If the assumption* A1 *holds*, *then the coefficient matrix of the SLEs* (3.3a)-(3.3c) *and* (3.4)

3.5 $$ W(x,H,\overline{\Lambda})\stackrel{\mathrm{def}}{=}\left ( \textstyle\begin{array}{@{}c@{\quad}c@{\quad}c@{}} H & \nabla\mathcal{A}(x)^{\mathrm{T}} & \nabla h(x) \\ (\overline{\Lambda}\otimes_{s}I_{m}) \nabla\mathcal{A}(x) & \mathcal{A}(x)\otimes_{s}I & 0 \\ \nabla h(x)^{\mathrm{T}} & 0 & 0 \end{array}\displaystyle \right ) $$

*is nonsingular*, *hence*, *SLEs* (3.3a)-(3.3c) *and* (3.4) *have a unique solution*, *respectively*.

The proof is elementary and it is omitted here.

In our algorithm the following exact penalty function is used as a merit function for line search:

3.6 $$ P(x;\sigma)=f(x)+\sigma\sum_{j\in\mathcal{ E}} \bigl\vert h_{j}(x) \bigr\vert , $$

where $\sigma>0$ is a penalty parameter. Further, we define a function $\overline{P}(\bullet ; d; \sigma):R^{n}\times R^{n}\times[0, +\infty )\rightarrow R$ associated with $P(x;\sigma)$ by

3.7 $$ \overline{P}(x;d;\sigma )=f(x)+\nabla f(x)^{\mathrm{T}}d+ \sigma\sum_{j\in\mathcal {E}} \bigl\vert h_{j}(x)+ \nabla h_{j}(x)^{\mathrm{T}}d \bigr\vert . $$

Now the algorithm is described in detail.

### Algorithm A

*Parameters*. $\alpha\in(0,\frac{1}{2})$, $\beta, \xi\in (0,1)$, $\lambda^{I}>0$, $\sigma_{{-1}}>0$, $\rho_{1}, \rho_{2}>0$.

*Initialization*. Select an initial iteration point $x^{0}\in \mathcal{F}_{0}$, $H_{0}\in\mathcal{S}_{++}^{n}$, $\overline{\Lambda}_{0}$ ($\in\mathcal{S}^{m}_{++}$) satisfying $\lambda^{I} I_{m}\preceq\overline {\Lambda}_{0} $ such that $\overline{\Lambda}_{0}$ and $\mathcal{A}(x^{0})$ commute. Let $\overline{\lambda}_{0}=\operatorname{svec}(\overline{\Lambda}_{0})$, $k:=0$.

Step 1.: Let $(d^{k0},\lambda^{k0}, \mu^{k0})$ be the solution of the SLE (3.3a)-(3.3c) in $(d, \lambda, \mu)$, *i.e.*,
  3.8 $$ \textstyle\begin{cases} {H_{k}}d+\nabla\mathcal{A}(x^{k})^{\mathrm{T}}\lambda+\sum_{j\in\mathcal {E}}{\mu_{j}}{\nabla h_{j}(x^{k})}=-\nabla f(x^{k}), \\ (\overline{\Lambda}_{k}\otimes_{s}I_{m})\nabla\mathcal{A}(x^{k})d+(\mathcal {A}(x^{k})\otimes_{s}I_{m})\lambda=0, \\ {\nabla h_{j}(x^{k})}^{\mathrm{T}}d=-h_{j}(x^{k}),\quad j\in\mathcal{E}. \end{cases} $$
  If $d^{k0}=0$, then stop, $x^{k}$ is a KKT point of NLSDP (1.1); else, go to Step 2.
Step 2.: Let $(d^{k1},\lambda^{k1}, \mu^{k1})$ be the solution of the SLE (3.4) in $(d, \lambda, \mu)$, *i.e.*,
  3.9 $$ \textstyle\begin{cases} {H_{k}}d+\nabla\mathcal{A}(x^{k})^{\mathrm{T}}\lambda+\sum_{j\in\mathcal {E}}{\mu_{j}}{\nabla h_{j}(x^{k})}=-\nabla f(x^{k}), \\ (\overline{\Lambda}_{k}\otimes_{s}I_{m})\nabla\mathcal{A}(x^{k})d+(\mathcal {A}(x^{k})\otimes_{s}I_{m})\lambda=-\overline{\lambda}_{k}\|d^{k0}\|, \\ {\nabla h_{j}(x^{k})}^{\mathrm{T}}d=-h_{j}(x^{k}),\quad j\in\mathcal{E}. \end{cases} $$
Step 3.: Compute the search direction $d^{k}$ and the approximate multiplier vector $(\lambda^{k}, \mu^{k})$:
  3.10 $$\begin{aligned} d^{k}=(1-\delta_{k})d^{k0}+ \delta_{k}d^{k1}, \end{aligned}$$
  3.11 $$\begin{aligned} \lambda^{k}=(1-\delta_{k})\lambda^{k0}+ \delta_{k}\lambda^{k1}, \end{aligned}$$
  3.12 $$\begin{aligned} \mu^{k}=(1-\delta_{k})\mu^{k0}+ \delta_{k}\mu^{k1}, \end{aligned}$$
  where
  3.13 $$ \delta_{k}= \textstyle\begin{cases} 1-\xi, \quad \mbox{if } \nabla f(x^{k})^{\mathrm{T}}d^{k1}\leq0; \\ 1,\hspace{25pt}\mbox{if } \nabla f(x^{k})^{\mathrm{T}}d^{k1}>0 \mbox{ and } \nabla f(x^{k})^{\mathrm{T}}d^{k1}\leq\nabla f(x^{k})^{\mathrm{T}}d^{k0}; \\ \min \{ \xi, \vert (1-\xi)\frac{\nabla f(x^{k})^{\mathrm{T}}d^{k0}+(\mu ^{k0})^{\mathrm{T}}h(x^{k})}{\nabla f(x^{k})^{\mathrm{T}}(d^{k0}-d^{k1})} \vert \} , \quad \mbox{otherwise}. \end{cases} $$
Step 4.: (Update the penalty parameter) Set $\overline {\sigma}_{k}=(3-\xi)\max_{j\in\mathcal{E}}|\mu_{j}^{k0}|+\rho _{1}$. The updating rule of $\sigma_{k}$ is as follows:
  3.14 $$ \sigma_{k}= \textstyle\begin{cases} \max\{\overline{\sigma}_{k}, \sigma_{{k-1}}+\rho _{2}\},& \mbox{if }\overline{\sigma}_{k}>\sigma_{{k-1}}, \\ \sigma_{{k-1}},&\mbox{otherwise}. \end{cases} $$
Step 5.: (Line search) Set the step size $t_{k}$ to be the first number of the sequence $\{1, \beta, \beta^{2},\ldots\}$ satisfying the following two inequalities:
  3.15 $$\begin{aligned}& P\bigl(x^{k}+td^{k};\sigma_{k}\bigr)\leq P \bigl(x^{k};\sigma_{k}\bigr)+t\alpha\bigl(\overline {P} \bigl(x^{k};d^{k};\sigma_{k}\bigr)-\overline{P} \bigl(x^{k};0;\sigma_{k}\bigr)\bigr), \end{aligned}$$
  3.16 $$\begin{aligned}& \mathcal{A}\bigl(x^{k}+td^{k}\bigr)\prec0. \end{aligned}$$
Step 6.: Set $x^{k+1}=x^{k}+t_{k}{d^{k}}$. Using the following methods to generate $\overline{\Lambda}_{k+1}$ commuting with $\mathcal{A}(x^{k+1})$:
  Step 6.1.: If the search direction $d^{k}$ does not descend or is not feasible, set $\overline{\Lambda}_{k+1}=I_{m}$ and go to Step 7.
  Step 6.2.: Compute the least eigenvalue $\lambda_{\mathrm{min}}(\overline{\Lambda}_{k})$ of the matrix $\bar{\Lambda}_{k}$. If $\lambda_{\mathrm{min}}(\overline{\Lambda}_{k})\geq\lambda^{I}$, then let $\overline{\Lambda}_{k+1}=\overline{\Lambda}_{k}$; otherwise, let $\overline{\Lambda}_{k+1}=\overline{\Lambda}_{k}+(\lambda^{I}-\lambda_{\mathrm{ min}}(\overline{\Lambda}_{k}))I_{m}$.
Step 7.: Set $\overline{\lambda} _{k+1}=\operatorname{svec}(\overline{\Lambda}_{k+1})$, and update $H_{k}$ by some method to $H_{k+1}$ such that $H_{k+1}$ is symmetric positive definite. Let $k:=k+1$, return to Step 1.

By (3.8), the following lemma is obvious.

### Lemma 3.3

*Suppose that the assumption* A1 *holds*. *If* $d^{k0}=0$, *then* $x^{k}$ *is a KKT point of NLSDP* (1.1).

### Lemma 3.4

*Suppose that the assumption* A1 *holds*. *Then the search direction* $d^{k}$ *of Algorithm* A *satisfies the following inequality*:

3.17 $$ \nabla f\bigl(x^{k}\bigr)^{\mathrm{T}}d^{k} \leq-\xi \bigl(d^{k0}\bigr)^{\mathrm{T}}H_{k}d^{k0}+(3- \xi)\sum_{j\in\mathcal{E}} \bigl\vert \mu _{j}^{k0}h_{j} \bigl(x^{k}\bigr) \bigr\vert . $$

### Proof

First we show that the inequality

3.18 $$ \nabla f\bigl(x^{k}\bigr)^{\mathrm{T}}d^{k0} \leq -\bigl(d^{k0}\bigr)^{\mathrm{T}}H_{k}d^{k0}+ \sum_{j\in\mathcal{E}} \bigl\vert \mu_{j}^{k0}h_{j} \bigl(x^{k}\bigr) \bigr\vert $$

holds. Premultiplying the first equation of (3.8) by $(d^{k0})^{\mathrm{T}}$, we obtain

3.19 $$ \bigl(d^{k0}\bigr)^{\mathrm{T}}H_{k}d^{k0}+ \sum_{j\in\mathcal{E}}\mu _{j}^{k0} \bigl(d^{k0}\bigr)^{\mathrm{T}}\nabla h_{j} \bigl(x^{k}\bigr)+\bigl(d^{k0}\bigr)^{\mathrm{T}}\nabla \mathcal {A}\bigl(x^{k}\bigr)^{\mathrm{T}}\lambda^{k0}=- \bigl(d^{k0}\bigr)^{\mathrm{T}}\nabla f \bigl(x^{k} \bigr). $$

According to the second equation of (3.8), we get

$$\bigl(d^{k0}\bigr)^{\mathrm{T}}\nabla\mathcal{A} \bigl(x^{k}\bigr)^{\mathrm{T}}\lambda^{k0}=-\bigl(\lambda ^{k0}\bigr)^{\mathrm{T}}\bigl((\overline{\Lambda}_{k} \otimes_{s}I_{m})^{-1}\bigl(\mathcal {A} \bigl(x^{k}\bigr)\otimes_{s}I_{m}\bigr) \bigr)^{\mathrm{T}}\lambda^{k0}. $$

Substituting the above equality and the third equality of (3.8) into (3.19), we have

$$\begin{aligned}& \bigl(d^{k0}\bigr)^{\mathrm{T}}\nabla f\bigl(x^{k} \bigr) \\& \quad =-\bigl(d^{k0}\bigr)^{\mathrm{T}}H_{k}d^{k0}+ \bigl(\lambda^{k0}\bigr)^{\mathrm{T}}\bigl((\overline{\Lambda }_{k}\otimes_{s}I_{m})^{-1}\bigl( \mathcal{A}\bigl(x^{k}\bigr)\otimes_{s}I_{m}\bigr) \bigr)^{\mathrm{T}}\lambda^{k0} +\sum_{j\in\mathcal{E}} \mu_{j}^{k0}h_{j}\bigl(x^{k}\bigr). \end{aligned}$$

In view of Lemma 2.4, the matrix $(\overline{\Lambda }_{k}\otimes_{s}I_{m})^{-1}(\mathcal{A}(x^{k})\otimes_{s}I_{m})$ is negative semidefinite, so it follows from the above equality that

$$ \bigl(d^{k0}\bigr)^{\mathrm{T}}\nabla f\bigl(x^{k} \bigr)\leq-\bigl(d^{k0}\bigr)^{\mathrm{T}}H_{k}d^{k0}+ \sum_{j\in\mathcal{E}} \bigl\vert \mu_{j}^{k0}h_{j} \bigl(x^{k}\bigr) \bigr\vert , $$

*i.e.*, the inequality (3.18) holds.

Next, we will prove the inequality (3.17) is true. The rest of the proof is divided into three cases.

*Case* A. $\nabla f(x^{k})^{\mathrm{T}}d^{k1}\leq0$. From (3.13) we have $\delta_{k}=1-\xi$. It follows from (3.10), (3.13), (3.18) and $\xi\in(0,1)$ that

3.20 $$\begin{aligned} \nabla f\bigl(x^{k}\bigr)^{\mathrm{T}}d^{k} \leq&-\xi\bigl(d^{k0}\bigr)^{\mathrm{T}}H_{k}d^{k0}+ \xi\sum_{j\in\mathcal{E}} \bigl\vert \mu _{j}^{k0}h_{j} \bigl(x^{k}\bigr) \bigr\vert \\ \leq&-\xi\bigl(d^{k0}\bigr)^{\mathrm{T}}H_{k}d^{k0}+(3- \xi)\sum_{j\in\mathcal {E}} \bigl\vert \mu_{j}^{k0}h_{j} \bigl(x^{k}\bigr) \bigr\vert , \end{aligned}$$

that is, (3.17) holds.

*Case* B. $\nabla f(x^{k})^{\mathrm{T}}d^{k1}>0$ and $\nabla f(x^{k})^{\mathrm{T}}d^{k1}\leq\nabla f(x^{k})^{\mathrm{T}}d^{k0}$. From (3.13), one has $\delta_{k}=1$. It follows from (3.10), (3.19) and $\xi \in(0,1)$ that

$$\begin{aligned} \nabla f\bigl(x^{k}\bigr)^{\mathrm{T}}d^{k} =& \nabla f\bigl(x^{k}\bigr)^{\mathrm{T}}d^{k1}\leq\nabla f\bigl(x^{k}\bigr)^{\mathrm{T}}d^{k0} \\ \leq& -\bigl(d^{k0}\bigr)^{\mathrm{T}}H_{k}d^{k0}+ \sum_{j\in\mathcal{E}} \bigl\vert \mu _{j}^{k0}h_{j} \bigl(x^{k}\bigr) \bigr\vert , \end{aligned}$$

which implies (3.17) holds.

*Case* C. $\nabla f(x^{k})^{\mathrm{T}}d^{k1}>0$ and $\nabla f(x^{k})^{\mathrm{T}}d^{k1}>\nabla f(x^{k})^{\mathrm{T}}d^{k0}$. It follows from (3.13) and $\xi\in(0,1)$ that

3.21 $$\begin{aligned} \delta_{k} =& \biggl\vert (1-\xi)\frac{\nabla f(x^{k})^{\mathrm{T}}d^{k0}+(\mu ^{k0})^{\mathrm{T}}h(x^{k})}{\nabla f(x^{k})^{\mathrm{T}}(d^{k1}-d^{k0})} \biggr\vert \\ \leq&\frac{|(\xi-1)\nabla f(x^{k})^{\mathrm{T}}d^{k0}|+|(\mu^{k0})^{\mathrm{T}}h(x^{k})|}{\nabla f(x^{k})^{\mathrm{T}}(d^{k1}-d^{k0})}. \end{aligned}$$

If $\nabla f(x^{k})^{\mathrm{T}}d^{k0}\leq0$, then we obtain from the above inequality

$$(1-\delta_{k})\nabla f\bigl(x^{k}\bigr)^{\mathrm{T}}d^{k0}+ \delta_{k}\nabla f\bigl(x^{k}\bigr)^{\mathrm{T}}d^{k1} \leq\xi\nabla f\bigl(x^{k}\bigr)^{\mathrm{T}}d^{k0}+ \bigl\vert \bigl(\mu^{k0}\bigr)^{\mathrm{T}}h \bigl(x^{k}\bigr) \bigr\vert , $$

which together with (3.10) and (3.18) gives

3.22 $$\begin{aligned} \nabla f\bigl(x^{k}\bigr)^{\mathrm{T}}d^{k} \leq&-\xi\bigl(d^{k0}\bigr)^{\mathrm{T}}H_{k}d^{k0}+(1+ \xi)\sum_{j\in\mathcal{E}} \bigl\vert \mu_{j}^{k0}h_{j} \bigl(x^{k}\bigr) \bigr\vert \\ \leq&-\xi\bigl(d^{k0}\bigr)^{\mathrm{T}}H_{k}d^{k0}+(3- \xi)\sum_{j\in\mathcal {E}} \bigl\vert \mu_{j}^{k0}h_{j} \bigl(x^{k}\bigr) \bigr\vert . \end{aligned}$$

If $\nabla f(x^{k})^{\mathrm{T}}d^{k0}>0$, then the inequality (3.21) gives rise to

$$\delta_{k}\nabla f\bigl(x^{k}\bigr)^{\mathrm{T}}d^{k1}- \delta_{k}\nabla f\bigl(x^{k}\bigr)^{\mathrm{T}}d^{k0} \leq(1-\xi)\nabla f\bigl(x^{k}\bigr)^{\mathrm{T}}d^{k0}+ \bigl\vert \bigl(\mu^{k0}\bigr)^{\mathrm{T}} h \bigl(x^{k}\bigr) \bigr\vert , $$

which together with (3.10) and (3.18) shows

3.23 $$\begin{aligned} \nabla f\bigl(x^{k}\bigr)^{\mathrm{T}}d^{k} \leq&-(2-\xi) \bigl(d^{k0}\bigr)^{\mathrm{T}}H_{k}d^{k0}+(3- \xi)\sum_{j\in\mathcal {E}} \bigl\vert \mu_{j}^{k0}h_{j} \bigl(x^{k}\bigr) \bigr\vert \\ \leq&-\xi\bigl(d^{k0}\bigr)^{\mathrm{T}}H_{k}d^{k0}+(3- \xi)\sum_{j\in\mathcal {E}} \bigl\vert \mu_{j}^{k0}h_{j} \bigl(x^{k}\bigr) \bigr\vert . \end{aligned}$$

The inequalities (3.22) and (3.23) indicate that the inequality (3.17) is true. □

### Lemma 3.5

*Suppose that the assumption* A1 *holds*. *If* $x^{k}$ ($\in\mathcal{F}$) *is not a KKT point of NLSDP* (1.1), *then*

3.24 $$ \overline{P}\bigl(x^{k};d^{k}; \sigma_{k}\bigr)-\overline {P}\bigl(x^{k};0; \sigma_{k}\bigr)< 0. $$

### Proof

From (3.8) and (3.9) we know that $(d^{k},\lambda ^{k},\mu^{k})$ is the solution of the following SLE:

3.25a $$\begin{aligned}& {H_{k}}d+\nabla\mathcal{A}\bigl(x^{k}\bigr)^{\mathrm{T}} \lambda+\sum_{j\in\mathcal {E}}{\mu_{j}} {\nabla h_{j}\bigl(x^{k}\bigr)}=-\nabla f\bigl(x^{k} \bigr), \end{aligned}$$

3.25b $$\begin{aligned}& (\overline{\Lambda}_{k}\otimes_{s}I_{m})\nabla \mathcal{A}\bigl(x^{k}\bigr)d+\bigl(\mathcal {A}\bigl(x^{k} \bigr)\otimes_{s}I_{m}\bigr)\lambda=-\delta_{k} \overline{\lambda}_{k} \bigl\Vert d^{k0} \bigr\Vert , \end{aligned}$$

3.25c $$\begin{aligned}& {\nabla h_{j}\bigl(x^{k}\bigr)}^{\mathrm{T}}d=-h_{j} \bigl(x^{k}\bigr), \quad j\in\mathcal{E}. \end{aligned}$$

From the definition (3.6) of the function $\overline {P}(x^{k};d^{k};\sigma_{k})$ and (3.25c), we have

3.26 $$\begin{aligned}& \overline{P}\bigl(x^{k};d^{k};\sigma_{k}\bigr)- \overline{P}\bigl(x^{k};0;\sigma _{k}\bigr) \\& \quad = \nabla f \bigl(x^{k}\bigr)^{\mathrm{T}}d^{k}-\sigma_{k} \sum_{j\in\mathcal {E}} \bigl\vert h_{j} \bigl(x^{k}\bigr) \bigr\vert \\& \quad \leq -\xi\bigl(d^{k0}\bigr)^{\mathrm{T}}H_{k}d^{k0}+(3- \xi)\sum_{j\in\mathcal {E}} \bigl\vert \mu_{j}^{k0}h_{j} \bigl(x^{k}\bigr) \bigr\vert -\sigma_{k}\sum _{j\in\mathcal {E}} \bigl\vert h_{j}\bigl(x^{k}\bigr) \bigr\vert \\& \quad \leq -\xi\bigl(d^{k0}\bigr)^{\mathrm{T}}H_{k}d^{k0}+ \Bigl((3-\xi)\max_{j\in\mathcal {E}} \bigl\vert \mu_{j}^{k0} \bigr\vert -\sigma_{k}\Bigr)\sum_{j\in\mathcal {E}} \bigl\vert h_{j}\bigl(x^{k}\bigr) \bigr\vert , \end{aligned}$$

the first inequality above is due to (3.17).

Since $x^{k}$ is not a KKT point of NLSDP (1.1), it implies from Step 1 of Algorithm A that $d^{k0}\neq0$, so $(d^{k0})^{\mathrm{T}}H_{k} d^{k0}>0$. On the other hand, it follows from the updating rule of $\sigma_{k}$ that $\sigma_{k}>(3-\xi)\max_{j\in\mathcal{E}}|\mu _{j}^{k0}|$, therefore, (3.26) gives rise to

$$ \overline{P}\bigl(x^{k};d^{k};\sigma_{k}\bigr)- \overline{P}\bigl(x^{k};0;\sigma_{k}\bigr)< 0, $$

that is, the inequality (3.24) holds. □

### Lemma 3.6

*Suppose that the assumption* A1 *holds*. *If Algorithm* A *does not stop at the current iterate* $x^{k}$, *then* (3.15) *and* (3.16) *are satisfied for* $t>0$ *small enough*, *so Algorithm* A *is well defined*.

### Proof

It follows from the Taylor expansion and (3.6) that

3.27 $$\begin{aligned}& P\bigl(x^{k}+td^{k};\sigma_{k}\bigr)-P \bigl(x^{k};\sigma_{k}\bigr) \\& \quad = t\nabla f\bigl(x^{k}\bigr)^{\mathrm{T}}d^{k}+ \sigma_{k}\sum_{j\in\mathcal {E}}\bigl( \bigl\vert h_{j}\bigl(x^{k}\bigr)+t\nabla h_{j} \bigl(x^{k}\bigr)^{\mathrm{T}}d^{k} \bigr\vert - \bigl\vert h_{j}\bigl(x^{k}\bigr) \bigr\vert \bigr)+o(t) \\& \quad = \overline{P}\bigl(x^{k};td^{k};\sigma_{k} \bigr)-\overline{P}\bigl(x^{k};0;\sigma_{k} \bigr)+o(t). \end{aligned}$$

The second equality above is due to (3.7). From the convexity of $\overline{P}(x^{k};d;\sigma_{k})$ for *d*, we obtain

3.28 $$ \overline{P}\bigl(x^{k};td^{k}; \sigma_{k}\bigr)-\overline{P}\bigl(x^{k};0; \sigma_{k}\bigr)\leq t\bigl(\overline{P}\bigl(x^{k};d^{k}; \sigma_{k}\bigr)-\overline{P}\bigl(x^{k};0; \sigma_{k}\bigr)\bigr), $$

which together with (3.27) and Lemma 3.4 gives for *t* small enough

$$P\bigl(x^{k}+td^{k};\sigma_{k}\bigr)-P \bigl(x^{k};\sigma_{k}\bigr)\leq t\alpha\bigl(\overline {P} \bigl(x^{k};d^{k};\sigma_{k}\bigr)-\overline{P} \bigl(x^{k};0;\sigma_{k}\bigr)\bigr), $$

where $\alpha\in(0,1)$. Hence, (3.15) holds for sufficiently small $t>0$.

In what follows, we prove (3.16) holds for sufficiently small $t>0$. Since $\mathcal{A}(x)$ is twice continuously differentiable function, it follows from Taylor expansion that

3.29 $$ \mathcal{A}\bigl(x^{k}+td^{k}\bigr)= \mathcal{A}\bigl(x^{k}\bigr)+tD\mathcal {A}\bigl(x^{k} \bigr)d^{k}+o(t)=\mathcal{A}\bigl(x^{k}\bigr)+O(t). $$

Note that the largest eigenvalue function $\lambda_{\mathrm{max}}(A)=\max_{\|v\|=1}v^{\mathrm{T}}Av$, we deduce from (3.29) and ${\mathcal{A}}(x^{k})\prec0$ that

$$ \lambda_{\mathrm{max}}\bigl(\mathcal{A}\bigl(x^{k}+td^{k} \bigr)\bigr) =\max_{\|v\|=1}\bigl\{ v^{\mathrm{T}}\mathcal{A} \bigl(x^{k}\bigr)v+v^{\mathrm{T}}O(t)v\bigr\} < 0 $$

for $0< t<1$ small enough, which implies (3.16) holds for $0< t<1$ small enough.

By summarizing the above discussions, we conclude that Algorithm A is well defined. □

## Global convergence

If Algorithm A terminates at $x^{k}$ after a finite number of iterations, we know from Lemma 3.3 that $x^{k}$ is a KKT point of NLSDP (1.1). In this section, without loss of generality, we assume that the sequence $\{x^{k}\}$ generated by Algorithm A is infinite. We will prove any accumulation point of $\{x^{k}\}$ is a stationary point or a KKT point of NLSDP (1.1), *i.e.*, Algorithm A is globally convergent. We first generalize the definition of stationary point for nonlinear programming defined in [16] to nonlinear semidefinite programming.

### Definition 4.1

Let $x\in R^{n}$, if there exist a matrix Λ ($\in S^{m}$) and a vector *μ* ($\in R^{l}$) such that

4.1 $$\begin{aligned}& \nabla_{x}L(x,\Lambda,\mu)=0, \end{aligned}$$

4.2 $$\begin{aligned}& \Lambda\mathcal{A}(x)=0,\qquad \mathcal{A}(x)\preceq0, \qquad h(x)=0, \end{aligned}$$

then *x* is called a stationary point of NLSDP (1.1).

In order to analyze the global convergence, some additional assumptions are required:

A2 *The sequence* $\{x^{k}\}$ *yielded by Algorithm* A *lies in a nonempty closed and bounded set* ${\mathcal{X}}$.

A3 *The functions* $f(x)$, $h(x)$ *and* ${\mathcal {A}}(x)$ *are twice continuously differentiable on an open set containing* ${\mathcal{X}}$.

A4 *There exists a positive constant* $\lambda^{s}$ *such that* $\lambda^{s}>\lambda^{I}$ *and* $\lambda^{I} I_{m}\preceq\overline{\Lambda}_{k}\preceq\lambda^{s}I_{m}$ *for all k.*

A5 *The matrix* $H_{k}$ *is uniformly positive definite, i.e., there exist two positive constants* *a* *and* *b* *such that* $a\|y\|^{2}\leq y^{\mathrm {T}}H_{k}y\leq b\|y\|^{2}$ *for all* $y\in R^{n}$ *.*

Let $x^{*}$ be an accumulation point of $\{x^{k}\}$, then there exists a subset $\mathcal{K}\subseteq\{1,2,\ldots\}$ such that $\lim_{k\in\mathcal{K}}x^{k}=x^{*}$. Without loss of generality, we suppose

$$\begin{aligned}& H_{k}\stackrel{\mathcal{K}}{\longrightarrow}H_{*},\qquad \nabla h \bigl(x^{k}\bigr) \stackrel{\mathcal{K}}{\longrightarrow}\nabla h\bigl(x^{*} \bigr), \\& \overline{\Lambda }_{k}\stackrel{\mathcal{K}}{\longrightarrow}\overline{\Lambda}_{*},\qquad W\bigl(x^{k}, H_{k}, \overline{\Lambda}_{k}\bigr) \stackrel{\mathcal{K}}{\longrightarrow}W\bigl(x^{*}, H_{*}, \overline{\Lambda}_{*}\bigr), \end{aligned}$$

where $W(x^{k}, H_{k}, \overline{\Lambda}_{k})$ is defined by (3.5) and

$$W\bigl(x^{*}, H_{*}, \overline{\Lambda}_{*}\bigr)\stackrel{\mathrm{def}}{=} \left ( \textstyle\begin{array}{@{}c@{\quad}c@{\quad}c@{}} H_{*} & \nabla\mathcal{A}(x^{*})^{\mathrm{T}} & \nabla h(x^{*}) \\ (\overline{\Lambda}_{*}\otimes_{s}I_{m})\nabla\mathcal{A}(x^{*}) & \mathcal{A}(x^{*})\otimes_{s}I_{m} & 0 \\ \nabla h(x^{*})^{\mathrm{T}} & 0 & 0 \end{array}\displaystyle \right ). $$

From the assumptions A2-A3, we obtain the following conclusions immediately.

### Lemma 4.1

*Suppose the assumptions* A2-A3 *hold*. *Then there exists a constant* $\overline{M}>1$ *such that* $|f(y^{k})|\leq \overline{M}$, $\|{\nabla}f(y^{k})\|\leq\overline{M}$, $\|{\nabla^{2}}f(y^{k})\|\leq\overline {M}$, $\|h(y^{k})\|\leq\overline{M}$, $\|{\nabla}h(y^{k})\|\leq\overline{M}$, $\|\mathcal{A}(y^{k})\|_{{F}}\leq \overline{M}$, $\|D\mathcal{A}(y^{k})\|_{{F}}\leq\overline{M}$ *and* $\|D^{2}\mathcal {A}(y^{k})\|_{{F}}\leq\overline{M}$, *for any* $y^{k}\in\mathcal{N}(x^{k})$, *where* $\mathcal{N}(x^{k})$ *is a neighborhood of* $x^{k}$.

### Lemma 4.2

*Suppose the assumptions* A1-A5 *hold*. *Then*

1. *there exists a constant* $c_{1}>0$ *such that* $\|W(x^{k}, H_{k}, \overline{\Lambda}_{k})^{-1}\|\leq c_{1}$ *for any* $k\in\mathcal{K}$;
2. *there exists a constant* $\widehat{M}>1$ *such that* $\|\lambda ^{k0}\|\leq\widehat{M}$, $\|\lambda^{k1}\|\leq\widehat{M}$, $\|\mu^{k0}\|\leq\widehat{M}$, $\|\mu^{k1}\|\leq\widehat{M}$, $\|d^{k0}\| \leq\widehat{M}$ *and* $\|d^{k1}\|\leq\widehat{M}$ *for any* $k\in \mathcal{K}$.

The following result is an important property of the penalty parameter $\sigma_{k}$, which is obtained by the updating rule (3.14).

### Lemma 4.3

*Suppose the assumptions* A1-A5 *hold*. *Then the penalty parameter* $\sigma_{k}$ *is updated only in a finite number of steps*.

Based on Lemma 4.3, in the rest of the paper, we assume, without loss of generality, that $\sigma_{k}\equiv\tilde {\sigma}$ for all *k*, where

$$ \tilde{\sigma}>\sup_{k}\Bigl\{ (3-\xi)\max _{j\in\mathcal {E}} \bigl\vert \mu^{k0}_{j} \bigr\vert \Bigr\} . $$

By using of Lemma 4.2, we obtain the following result.

### Lemma 4.4

*Suppose the assumptions* A1-A5 *hold*. *Then there exists a constant* $c_{2}>0$ *such that*

4.3 $$ \bigl\Vert d^{k}-d^{k0} \bigr\Vert \leq c_{2} \bigl\Vert d^{k0} \bigr\Vert . $$

For the sake of simplicity, in the rest of this section, let $(d^{*0}, \mu^{*0}, \lambda^{*0})$ be the solution of the following SLE in $(d, \mu, \lambda)$:

4.4 $$ \textstyle\begin{cases} {H_{*}}d+\nabla\mathcal{A}(x^{*})^{\mathrm{T}}\lambda+\sum_{j\in\mathcal {E}}{\mu_{j}}{\nabla h_{j}(x^{*})}=-\nabla f(x^{*}), \\ (\overline{\Lambda}_{*}\otimes_{s}I_{m})\nabla\mathcal{A}(x^{*})d+(\mathcal {A}(x^{*})\otimes_{s}I_{m})\lambda=0, \\ {\nabla h_{j}(x^{*})}^{\mathrm{T}}d=-h_{j}(x^{*}),\quad j\in\mathcal{E}. \end{cases} $$

Let $(d^{*1}, \mu^{*1}, \lambda^{*1})$ be the solution of the following SLE in $(d, \mu, \lambda)$:

4.5 $$ \textstyle\begin{cases} {H_{*}}d+\nabla\mathcal{A}(x^{*})^{\mathrm{T}}\lambda+\sum_{j\in\mathcal {E}}{\mu_{j}}{\nabla h_{j}(x^{*})}=-\nabla f(x^{*}), \\ (\overline{\Lambda}_{*}\otimes_{s} I_{m})\nabla\mathcal{A}(x^{*})d+(\mathcal {A}(x^{*})\otimes_{s} I_{m})\lambda=-\overline{\lambda}_{*}\|d^{*0}\|, \\ {\nabla h_{j}(x^{*})}^{\mathrm{T}}d=-h_{j}(x^{*}),\quad j\in\mathcal{E}. \end{cases} $$

From the above equalities and Lemma 4.2, we obtain the following conclusion.

### Lemma 4.5

*Suppose the assumptions* A1-A5 *hold*, *and* $\delta_{k}\stackrel{\mathcal{K}}{\longrightarrow}\delta_{*}$. *Then*

(i) $d^{k0}\stackrel{\mathcal{K}}{\longrightarrow}d^{*0}$, $\mu ^{k0}\stackrel{\mathcal{K}}{\longrightarrow}\mu^{*0}$, $\lambda ^{k0}\stackrel{\mathcal{K}}{\longrightarrow}\lambda^{*0}$,
(ii) $d^{k1}\stackrel{\mathcal{K}}{\longrightarrow}d^{*1}$, $\mu ^{k1}\stackrel{\mathcal{K}}{\longrightarrow}\mu^{*1}$, $\lambda ^{k1}\stackrel{\mathcal{K}}{\longrightarrow}\lambda^{*1}$,
(iii) $d^{*0}=0$ *if and only if* $d^{*}=0$ *where* $d^{*}=(1-\delta _{*})d^{*0}+\delta_{*}d^{*1}$.

### Remark 4.1

By (3.13), we know that $\{\delta_{k}\}$ is bounded, so in the rest of the paper, we assume, without loss of generality, that $\delta_{k}\stackrel{\mathcal{K}}{\longrightarrow}\delta_{*}$.

### Lemma 4.6

*Suppose the assumptions* A1-A5 *hold*. *Let* $x^{*}$ *be an accumulation point of the sequence* $\{x^{k}\}$ *and* $x^{k}\stackrel{\mathcal{K}}{\longrightarrow}x^{*}$. *If* $d^{k}\stackrel{\mathcal{K}}{\longrightarrow}0$, *then* $x^{*}$ *is a KKT point or a stationary point of NLSDP* (1.1), *and* $\lambda^{k}\stackrel {\mathcal{K}}{\longrightarrow}\operatorname{svec}(\Lambda^{*})$, $\mu^{k}\stackrel {\mathcal{K}}{\longrightarrow}\mu^{*}$, *where* $(\Lambda^{*}, \mu^{*})$ *is the Lagrangian multiplier corresponding to* $x^{*}$.

### Proof

It is clear from Lemma 4.2 that $\{\lambda^{k}\}$ and $\{\mu^{k}\}$ are bounded. Assume that *λ̂*, *μ̂* are accumulation points of $\{\lambda^{k}\}$ and $\{\mu^{k}\}$, respectively. Without loss of generality, we assume that $\lambda^{k}\stackrel{\mathcal{K}}{\longrightarrow}\hat{\lambda}$ and $\mu^{k}\stackrel{\mathcal{K}}{\longrightarrow}\hat{\mu}$.

Obviously, $(d^{k}, \lambda^{k}, \mu^{k})$ satisfies the SLE (3.25a)-(3.25c). By taking the limit on $\mathcal{K}$ in (3.25a)-(3.25c), we obtain

4.6a $$\begin{aligned}& \nabla\mathcal{A}\bigl(x^{*}\bigr)\hat{\lambda}+\sum_{j\in\mathcal {E}} \hat{\mu}_{j}{\nabla h_{j}\bigl(x^{*}\bigr)}=-\nabla f \bigl(x^{*}\bigr), \end{aligned}$$

4.6b $$\begin{aligned}& \bigl(\mathcal{A}\bigl(x^{*}\bigr)\otimes_{s}I\bigr)\hat{\lambda }=0, \end{aligned}$$

4.6c $$\begin{aligned}& h_{j}\bigl(x^{*}\bigr)=0, \quad j\in\mathcal{E}. \end{aligned}$$

If $x^{*}\in\mathcal{F}_{0}$, *i.e.*, $\mathcal{A}(x^{*})\prec0$, then we know from Lemma 2.1(4) that $\mathcal{A}(x^{*})\otimes_{s}I$ is nonsingular, so the equation (4.6b) has a unique solution $\hat{\lambda}=0$. Let $\widehat{\Lambda}:=\operatorname{smat}(\hat {\lambda})=0$, so $\widehat{\Lambda}\mathcal{A}(x^{*})=0$. Together with (4.6a) and (4.6c), we conclude that $x^{*}$ is a KKT point of NLSDP (1.1).

If $x^{*}\in\Omega\backslash{\mathcal{F}}_{0}$, let $\widehat{\Lambda }:=\operatorname{smat}(\hat{\lambda})$. It follows from (4.6b) that $\operatorname{sym}(\widehat{\Lambda}\mathcal{A}(x^{*}))=0$, which means that $\widehat{\Lambda}\mathcal{A}(x^{*})$ is a skw-symmetric matrix. Hence $\operatorname{Tr}(\widehat{\Lambda}\mathcal{A}(x^{*}))=0$. According to Remark 2.2, we obtain $\widehat{\Lambda}\mathcal{A}(x^{*})=0$. Combining with (4.6a) and (4.6c), $x^{*}$ is a stationary point of NLSDP (1.1). $(\lambda^{*}, \mu^{*})$ is the Lagrangian multiplier corresponding to $x^{*}$, that is,

$$\begin{aligned}& \nabla\mathcal{A}\bigl(x^{*}\bigr)^{\mathrm{T}} {\lambda^{*}}+\sum _{j\in\mathcal {E}}{\mu^{*}_{j}} {\nabla h_{j}\bigl(x^{*} \bigr)}=-\nabla f\bigl(x^{*}\bigr), \\& \Lambda^{*}\mathcal{A}\bigl(x^{*}\bigr)=0, \end{aligned}$$

where $\Lambda^{*}=\operatorname{smat}(\lambda^{*})$. It is not difficult to verify that $(\lambda^{*}, \mu^{*})$ is the solution of the following SLE:

4.7a $$\begin{aligned}& \nabla\mathcal{A}\bigl(x^{*}\bigr)^{\mathrm{T}} {\lambda^{*}}+\sum _{j\in\mathcal {E}}{\mu^{*}_{j}} {\nabla h_{j}\bigl(x^{*} \bigr)}=-\nabla f\bigl(x^{*}\bigr), \end{aligned}$$

4.7b $$\begin{aligned}& \bigl(\mathcal{A}\bigl(x^{*}\bigr)\otimes_{s}I\bigr){ \lambda^{*}}=0. \end{aligned}$$

From (4.6a)-(4.6c), we know that $(\hat{\lambda}, \hat{\mu})$ is also the solution of (4.7a)-(4.7b). It is clear from the assumption A1 that the solution of (4.7a)-(4.7b) is unique, therefore, $\hat{\lambda}={\lambda^{*}}$, $\hat{\mu}=\mu^{*}$. The proof is completed. □

Based on Lemma 4.6, the following conclusion is obvious.

### Lemma 4.7

*Suppose the assumptions* A1-A5 *hold*. *Let* $x^{k}\stackrel{\mathcal{K}}{\longrightarrow}x^{*}$. *If* $d^{k-1}\stackrel{\mathcal{K}}{\longrightarrow}0$, *then* $x^{*}$ *is a KKT point or a stationary point of NLSDP* (1.1).

### Lemma 4.8

*Suppose the assumptions* A1-A5 *hold*, $x^{k} \stackrel{\mathcal{K}}{\longrightarrow}x^{*}$. *If* $\inf_{\mathcal{K}}\{\|d^{k-1}\|\}>0$, *then* $d^{k} \stackrel{\mathcal{K}}{\longrightarrow}0$.

### Proof

By contradiction, we assume that there exist a subset $\mathcal{K}'\subset\mathcal{K}$ and a constant $\bar{d}>0$ such that $\|d^{k}\|\geq\bar{d}$, $\forall k\ (\in{\mathcal{K}}')$ large enough. From the assumptions A1-A5, (3.13) and the updating rule of $\overline{\Lambda}_{k}$, we assume without loss of generality that $H_{k}\stackrel{\mathcal{K}'}{\longrightarrow}H_{*}$, $\delta_{k}\stackrel{\mathcal{K}'}{\longrightarrow}\delta_{*}$, $\overline {\Lambda}_{k}\stackrel{\mathcal{K}'}{\longrightarrow}\overline{\Lambda}_{*}$. On the other hand, it follows from the updating rule of $\overline {\Lambda}_{k}$ and the assumption A4 that $\overline{\Lambda}_{*}$ is positive definite. According to Lemma 4.5(iii), there exists $\underline{d}>0$ such that $\|d^{k0}\|\geq\underline{d}$ for all $k\in\mathcal{K}'$.

Firstly, we show that there exists $\underline{t}>0$ independent of *k* such that (3.15) and (3.16) are satisfied for all $t\geq\underline{t}$. For any $k\in\mathcal{K}'$, it is clear from the assumptions A1 and A5 and Lemmas 3.3-3.4 and Lemmas 4.1-4.2 that

4.8 $$ \overline{P}\bigl(x^{k};d^{k};\tilde{\sigma} \bigr)-\overline{P}\bigl(x^{k};0;\tilde {\sigma}\bigr) \leq-\xi a \underline{d}^{2}. $$

Together with (3.27)-(3.28), there exists $t_{f}>0$ independent of *k* such that

4.9 $$ P\bigl(x^{k}+td^{k};\tilde{\sigma}\bigr)-P \bigl(x^{k};\tilde{\sigma}\bigr)\leq t\alpha \bigl[\overline{P} \bigl(x^{k};d^{k};\tilde{\sigma}\bigr)-\overline{P} \bigl(x^{k};0;\tilde {\sigma}\bigr)\bigr] $$

for all $k\in\mathcal{K}'$ and $t\in(0,t_{f}]$, where $\alpha\in (0,1)$. The above inequality shows the inequality (3.15) holds.

We next prove the inequality (3.16) holds. It follows from (3.8) and Lemma 2.1(4) and Lemma 2.4 that

$$\begin{aligned}& \bigl\vert \nabla f\bigl(x^{k}\bigr)^{\mathrm{T}}d^{k0}+ \bigl(\mu^{k0}\bigr)^{\mathrm{T}}h\bigl(x^{k}\bigr) \bigr\vert \\& \quad = \bigl\vert -\bigl(d^{k0}\bigr)^{\mathrm{T}}H_{k}d^{k0}+ \bigl(\lambda^{k0}\bigr)^{\mathrm{T}}\bigl((\overline { \Lambda}_{k}\otimes_{s}I_{m})^{-1}\bigl( \mathcal{A}\bigl(x^{k}\bigr)\otimes_{s}I_{m}\bigr) \bigr)^{\mathrm{T}}\lambda^{k0} \bigr\vert \\& \quad \geq a \bigl\Vert d^{k0} \bigr\Vert ^{2}. \end{aligned}$$

Combining with Lemmas 4.1-4.2 and (3.13), there exists a constant $0< \tilde{\delta} \leq1$ such that $\delta_{k}\geq\tilde{\delta}$ for $k\in\mathcal{K}'$. By the mean-value theorem and Lemmas 4.1-4.2, we obtain

4.10 $$\begin{aligned} \mathcal{A}\bigl(x^{k}+td^{k}\bigr) =& \mathcal{A}\bigl(x^{k}\bigr)+tD\mathcal {A}\bigl(x^{k} \bigr)d^{k}+t^{2}\bigl(D^{2}\mathcal{A}\bigl(x+t \vartheta d^{k}\bigr) \bigl(d^{k},d^{k}\bigr)\bigr) \\ \preceq&\mathcal{A}\bigl(x^{k}\bigr)+tD\mathcal{A} \bigl(x^{k}\bigr)d^{k}+t^{2}M^{3}I_{m} \end{aligned}$$

for any $k\in\mathcal{K}'$, where $\vartheta\in(0,1)$, $M={\mathrm{max}}\{ \widehat{M}, \overline{M}\}$. Let $N(t;x^{k})=\mathcal{A}(x^{k})+tD\mathcal {A}(x^{k})d^{k}+t^{2}M^{3}I_{m}$, the above inequality is rewritten as

4.11 $$ \mathcal{A}\bigl(x^{k}+td^{k}\bigr)\preceq N \bigl(t;x^{k}\bigr), $$

thus, in order to prove that $\mathcal{A}(x^{k}+td^{k}) $ is negative definite, it is sufficient to prove that $N(t; x^{k}) $ is negative definite. In view of $\overline{\Lambda}_{k}\succ0$, the definition (2.2) of sym and Lemma 2.2, it is sufficient to show that there exists $t_{{\mathcal{A}}}>0$ independent of *k* such that

4.12 $$ \operatorname{sym}\bigl(\overline{\Lambda}_{k}N \bigl(t;x^{k}\bigr)\bigr)\prec 0, \quad \forall t\in (0,t_{{\mathcal{A}}} ]. $$

In view of (2.10), (2.5) and Lemma 2.1(1), we obtain

4.13 $$ (\overline{\Lambda}_{k}\otimes_{s}I_{m}) \nabla\mathcal{A}\bigl(x^{k}\bigr)d^{k} =\operatorname{svec} \bigl(\operatorname{sym}\bigl(\overline{\Lambda}_{k}D\mathcal{A} \bigl(x^{k}\bigr)d^{k}\bigr)\bigr). $$

Let $\Lambda^{k}=\operatorname{smat}(\lambda^{k})$, *i.e.*, $\lambda^{k}=\operatorname{svec}(\Lambda^{k})$, it is obvious from (2.5) that

4.14 $$ \bigl(\mathcal{A}\bigl(x^{k}\bigr)\otimes_{s}I_{m} \bigr)\lambda^{k} = \bigl(\mathcal{A}\bigl(x^{k}\bigr)\otimes _{s}I_{m}\bigr)\operatorname{svec}\bigl(\Lambda^{k} \bigr) = \operatorname{svec}\bigl(\operatorname{sym}\bigl(\Lambda^{k} \mathcal{A}\bigl(x^{k}\bigr)\bigr)\bigr). $$

Hence, (4.13), (4.14) and (3.25b) give rise to

$$\begin{aligned}& \operatorname{sym}\bigl(\overline{\Lambda}_{k}D\mathcal{A} \bigl(x^{k}\bigr)d^{k}+\Lambda^{k}\mathcal {A} \bigl(x^{k}\bigr)\bigr) \\& \quad = \operatorname{smat}\bigl(\operatorname{svec}\bigl(\operatorname{sym} \bigl(\overline{\Lambda}_{k}D\mathcal {A}\bigl(x^{k} \bigr)d^{k}\bigr)\bigr)+\operatorname{svec}\bigl(\operatorname{sym} \bigl(\Lambda^{k}\mathcal{ A}\bigl(x^{k}\bigr)\bigr)\bigr) \bigr) \\& \quad = \operatorname{smat}\bigl(-\delta_{k}\overline{ \lambda}_{k} \bigl\Vert d^{k0} \bigr\Vert \bigr)=- \delta_{k} \bigl\Vert d^{k0} \bigr\Vert \overline{ \Lambda}_{k}. \end{aligned}$$

Based on the above equality, we have

4.15 $$\begin{aligned} \operatorname{sym}\bigl(\overline{\Lambda}_{k}N\bigl(t;x^{k} \bigr)\bigr) =& \operatorname{sym}\bigl(\overline{\Lambda }_{k}\bigl( \mathcal{A}\bigl(x^{k}\bigr)+tD\mathcal{A}\bigl(x^{k} \bigr)d^{k}+t^{2}M^{3}I_{m}\bigr)\bigr) \\ =& \operatorname{sym}\bigl(\bigl(\overline{\Lambda}_{k}-t \Lambda^{k}\bigr)\mathcal {A}\bigl(x^{k}\bigr)\bigr)+ \bigl(t^{2}M^{3}\overline{\Lambda}_{k}-t \delta_{k} \bigl\Vert d^{k0} \bigr\Vert \overline { \Lambda}_{k}\bigr) \\ \prec& \operatorname{sym}\bigl(\bigl(\overline{\Lambda}_{k}-t \Lambda^{k}\bigr)\mathcal {A}\bigl(x^{k}\bigr)\bigr)+ \bigl(2t^{2}M^{3}-t\tilde{\delta}\underline{d}\bigr) \overline{\Lambda}_{k}; \end{aligned}$$

note the positive definiteness of $\overline{\Lambda}_{k} $, hence, if

4.16 $$ \max\bigl\{ v^{\mathrm{T}}\bigl(\bigl(\overline{\Lambda }_{k}-t\Lambda^{k}\bigr)\mathcal{A}\bigl(x^{k} \bigr)\bigr)v: v\in R^{m}, \|v\|=1\bigr\} \leq0, \quad \mbox{for any } k \in\mathcal{K}', $$

then (4.12) holds for $t\leq\frac{\tilde{\delta}\underline{d}}{2M^{3}}$.

Since $\overline{\Lambda}_{k}$ and $\mathcal{A}(x^{k})$ are symmetric and commuting, there exists an orthogonal matrix $Q_{k}$ such that

4.17 $$ \overline{\Lambda}_{k}=Q_{k}^{\mathrm{T}} \overline{D}_{\lambda}^{k}Q_{k}, \qquad \mathcal{A} \bigl(x^{k}\bigr)=Q_{k}^{\mathrm{T}}D_{{\mathcal{A}}}^{k}Q_{k}, $$

where $\overline{D}_{\lambda}^{k}$ and $D_{{\mathcal{A}}}^{k}$ are diagonal matrices. Then $(\overline{\Lambda}_{k}-t\Lambda^{k})\mathcal{A}(x^{k}) = Q^{\mathrm{T}}_{k}(\overline{D}_{\lambda}^{k}-tQ_{k}\Lambda^{k} Q^{\mathrm{T}}_{k}) D_{{\mathcal{A}}}^{k}Q_{k}$. Let $\widetilde{\Lambda}^{k}=Q_{k}\Lambda ^{k}Q_{k}^{\mathrm{T}}$, so in order to prove (4.16), it is enough to show that there exists a constant $t_{{\mathcal{A}}}>0$ such that

4.18 $$ v^{\mathrm{T}}\bigl(\bigl(\overline{D}_{\lambda}^{k}-t \widetilde{\Lambda}^{k}\bigr)D^{k}_{{\mathcal {A}}}\bigr)v\leq0, \quad \forall v\mbox{: } \|v\|=1, $$

for any $t\in(0, t_{{\mathcal{A}}})$ and $k\in\mathcal{K}'$. By Lemma 4.6 and $\Lambda^{k}=\operatorname{smat}(\lambda^{k})$, we know $\{ \Lambda^{k}\}$ is bounded, furthermore, $\{\widetilde{\Lambda}^{k}\}$ is also bounded. Let $\widetilde{\Lambda}^{*}$ be an accumulation point of $\{\widetilde{\Lambda}^{k}\}$. Without loss of generality, we assume that $\widetilde{\Lambda}^{k}\stackrel{\mathcal{K}'}{\longrightarrow}\widetilde {\Lambda}^{*}$. Let $B^{k}=\widetilde{\Lambda}^{k}-\widetilde{\Lambda}^{*}$, obviously, $B^{k} \stackrel{{\mathcal{K}}'}{\longrightarrow}0$, thus there exists $\gamma>0$ such that

4.19 $$ \bigl\vert v^{\mathrm{T}}\bigl(B^{k}D^{k}_{{\mathcal{A}}} \bigr)v \bigr\vert < \gamma $$

for any $k\in\mathcal{K}'$. Note that

4.20 $$ v^{\mathrm{T}}\bigl(\overline{D}_{\lambda}^{k}-t \widetilde{\Lambda}^{k}\bigr)D^{k}_{{\mathcal{A}}}v =v^{\mathrm{T}}\bigl(\overline{D}_{\lambda}^{k}-t\widetilde{ \Lambda}^{*}\bigr)D^{k}_{{\mathcal {A}}}v-tv^{\mathrm{T}} \bigl(B^{k}D^{k}_{{\mathcal{A}}}\bigr)v. $$

It follows from the assumption A4 that all eigenvalues of $\overline {D}_{\lambda}^{k}$ are between $\lambda^{I}$ and $\lambda^{s}$ for all *k*. According to Weyl’s theorem (see [6]), there exists $t_{1}>0$ such that all eigenvalues of $(\overline{D}_{\lambda}^{k}-t\widetilde{\Lambda}^{*})$ are positive for any $t\in(0,t_{1}]$. We also know from ${\mathcal{A}}(x^{k}) \prec0$ and the second equality in (4.17) that $D_{{\mathcal{A}}}$ is negative definite. Therefore, for any *v* with $\|v\|=1$ and $t\in(0,t_{1}]$, it follows from Lemma 2.3 that $(\overline {D}_{\lambda}^{k}-t\widetilde{\Lambda}^{*})D_{{\mathcal{A}}}^{k}$ is also negative definite. Combining with (4.19), for any *v* with $\|v\|=1$ and any $t\in(0, t_{1})$, we obtain

4.21 $$ v^{\mathrm{T}}\bigl(\bigl(\overline{D}_{\lambda}^{k}-t\widetilde{\Lambda}^{*}\bigr)D_{{\mathcal{A}}}^{k} \bigr)v-tv^{\mathrm{T}}\bigl(B^{k}D^{k}_{{\mathcal{A}}} \bigr)v\leq0, $$

together with (4.20) shows that (4.18) is satisfied, further, (4.16) and (4.12) hold.

Let $t_{{\mathcal{A}}}={\mathrm{min}}\{t_{1}, \frac{m\underline{d}}{2M^{3}}\}$, thus (4.12) holds for any $t\in (0,t_{{\mathcal{A}}}]$. Hence, we see that $\mathcal{A}(x^{k}+td^{k})\prec0 $ holds for $t\in(0, t_{{\mathcal{A}}}]$ and any $k\in\mathcal{K}'$. Let $\bar{t}={\mathrm{min}}\{t_{f},t_{{\mathcal{A}}}\}$, for any $\underline {t}\in(0,\bar{t}]$, (3.15) and (3.16) are satisfied for all $t\geq\underline{t}$. Combining with (4.8) and (4.9), we obtain for any $k\in{\mathcal{K}}'$

4.22 $$ P\bigl(x^{k+1};\tilde{\sigma}\bigr)\leq P \bigl(x^{k};\tilde{\sigma}\bigr)-\underline {t}\alpha\xi a \underline{d}^{2}. $$

On the other hand, the sequence $\{P(x^{k};\tilde{\sigma})\}$ decreases monotonically and $P(x^{k};\tilde{\sigma})\stackrel{\mathcal{K}'}{\longrightarrow}P(x^{*};\tilde{\sigma})$, so $\{P(x^{k};\tilde{\sigma})\}^{\infty}_{k=1}$ is convergent. Let $\lim_{k\rightarrow\infty}P(x^{k};\tilde{\sigma})=\varrho$ and taking the limit in the above inequality, we have $-\underline{t}\xi \alpha a\underline{d}^{2}\geq0$, which is a contradiction. Hence, $d^{k}\stackrel{\mathcal{K}}{\longrightarrow}0$. □

Based on Lemmas 4.6-4.8, the following global convergence of Algorithm A is immediate.

### Theorem 4.1

*Suppose the assumptions* A1-A5 *hold*. *Then Algorithm* A *either terminates in a finite number of iterations at a KKT point of the NLSDP* (1.1), *or it generates a sequence* $\{x^{k}\}$ *whose every accumulation point is a KKT point or a stationary point of the NLSDP* (1.1).

## Numerical experiments

Algorithm A has been implemented in Matlab 2011b and the codes have been run on a 3.40 GHz Intel(R) Core(TM)i3-3240 machine with a Windows 7 system. We choose $H_{0}$ as *n*-order identical matrix and at each iteration, $H_{k}$ is updated by the damped BFGS formula in [15] and $\overline{\Lambda}_{0}$ as *m*-order identical matrix. In the numerical experiments, we choose the parameters as follows:

$$\begin{aligned}& \alpha=0.25,\qquad \beta=0.5,\qquad \xi=0.5,\qquad \lambda^{I}=0.5, \\& \sigma _{-1}=0.5, \qquad \rho_{1}=1, \qquad \rho_{2}=2. \end{aligned}$$

The stop criterion is $\|d^{k0}\|\leq10^{-4}$.

The test problems are described as follows:

I. The first test problem is Rosen-Suzuki problem [29] combined with a negative semidefinite constraint and denoted by *CM*:

$$\begin{aligned}& \min f_{0}(x) = x_{1}^{2}+x_{2}^{2}+2x_{3}^{2}+x_{4}^{2}-5x_{1}-5x_{2}-21x_{3}+7x_{4} \\& \quad \mbox{s.t. } x_{1}^{2}+x_{2}^{2}+x_{3}^{2}+x_{4}^{2}+x_{1}-x_{2}+x_{3}-x_{4}-8=0, \\& \hphantom{\quad \mbox{s.t.}}\ x_{1}^{2}+2x_{2}^{2}+x_{3}^{2}+2x_{4}^{2}-x_{1}-x_{4}-9=0, \\& \hphantom{\quad \mbox{s.t.}}\ 2x_{1}^{2}+x_{2}^{2}+x_{3}^{2}+2x_{1}-x_{2}-x_{4}-5=0, \\& \hphantom{\quad \mbox{s.t.}}\ \left( \textstyle\begin{array}{@{}c@{\quad}c@{\quad}c@{\quad}c@{}} -x_{2}-x_{3} & 0 & 0 & 0 \\ 0 & -2x_{4} & -x_{1} & 0 \\ 0 &-x_{1} & -2x_{4} & 0 \\ 0 & 0 & 0 & -x_{2}-x_{3} \end{array}\displaystyle \right)\preceq0. \end{aligned}$$

II. We select some test problems from [7] only with equality constraints and we add a negative semidefinite matrix constraint.

1. We select the problems HS6, HS7, HS8, HS9 combined with the following $2\times2$ order symmetric matrix which comes from [14] and rename them MHS6, MHS7, MHS8 and MHS9, respectively:
  $$\left( \textstyle\begin{array}{@{}c@{\quad}c@{}} -x_{1}^{2} & -\frac{x_{1}}{2} \\ -\frac{x_{1}}{2} &-x_{2}^{2} \end{array}\displaystyle \right)\preceq0. $$
2. Choose the problems HS26, HS27, HS28 and HS61 combined with the following $3\times3$ order symmetric matrix and rename them MHS26, MHS27, MHS28 and MHS61, respectively:
  $$\left( \textstyle\begin{array}{@{}c@{\quad}c@{\quad}c@{}} -x_{1}^{2} & -\frac{x_{1}}{2} & 0 \\ -\frac{x_{1}}{2}&-x_{2}^{2} & 0 \\ 0 & 0 & -x_{3}^{4} \end{array}\displaystyle \right)\preceq0. $$
3. Choose the problems HS40, HS42, HS47, HS48, HS50, HS51, HS77 and HS79, adding the negative semidefinite matrix constraint in the problem *CM* and renaming them MHS40, MHS42, MHS47, MHS48, MHS50, MHS51, MHS77 and MHS79.

III. Nearest correlation matrix problem (NCM for short) (see [23]):

$$\begin{aligned}& \min f(X) = \frac{1}{2}\|X-A\|_{{F}} \\& \quad \mbox{s.t. } X\succeq\epsilon I, \\& \hphantom{\quad \mbox{s.t.}}\ X_{ii}=1,\quad i=1,2,\ldots,m, \end{aligned}$$

where $A\in\mathcal{S}^{m}$ is given. In NCM problem, eigenvalues of *X* should not be less than *ϵ*, and the diagonal elements of *X* are equal to 1. Elements of the matrix *A* are uniform random numbers in $[-1,1]$ with $A_{ii}=1$, $i=1,2,\ldots,m$. Set $\epsilon=10^{-3}$. In addition, we compare with the results of [23] (Algo. SDPIP for short) and [24] (Algo. YYNY for short), and their results from [24].

The numerical results are listed in Table 1 and Table 2. The meanings of the notations in Table 1 and Table 2 are as follows:

- *n*: the number of variables;
- *l*: the number of equality constraints;
- *m*: the dimensionality of the negative semidefinite matrix;
- Iter.: the number of iterations;
- NF: the number of evaluations for $f(x)$;
- NC: the number of evaluations for all constraint functions;
- $f_{\mathrm{final}}$: the optimal value;
- Time (s): the time of calculation;
-: means that the result is not given.

**Table 1:** **The numerical results of test problems I and II**

**Problem**	***n***	***l***	***m***	$\boldsymbol{x^{0}}$	**Iter.**	**NF**	**NC**	$\boldsymbol{f_{\mathrm {final}}}$	**Time (s)**
CM	4	3	4	$(2.5, 2.5, 2.5, -2.5)^{\mathrm{T}}$	19	72	72	−4.400000e + 001	4.097408e − 001
PHS6	2	1	2	$(-2, -2)^{\mathrm{T}}$	99	128	128	1.226381e − 006	3.541575e − 001
PHS7	2	1	2	$(1,5)^{\mathrm{T}}$	43	169	169	−1.732051e + 000	3.551911e − 001
PHS8	2	2	2	$(1,4)^{\mathrm{T}}$	4	4	4	−1	2.195229e − 001
PHS9	2	1	2	$(-4,4)^{\mathrm{T}}$	2	2	2	−4.999996e − 001	2.025914e − 001
PHS26	3	1	3	$(1.5,1.5,1.5)^{\mathrm{T}}$	28	28	28	3.726010e − 005	2.514937e − 001
PHS27	3	1	3	$(-1,1,1)^{\mathrm{T}}$	17	17	17	5.426241e − 002	2.354974e − 001
PHS28	3	1	3	$(1,-1,-1)^{\mathrm{T}}$	6	6	6	6.756098e − 001	1.708627e − 001
PHS40	4	3	4	$(0.5,0.5,0.5,0.5)^{\mathrm{T}}$	8	10	10	−2.500001e − 001	2.773717e − 001
PHS42	4	2	4	$(-1,1,1,1)^{\mathrm{T}}$	17	28	28	1.385766e + 001	2.415490e − 001
PHS47	5	3	4	$(-1,1,1,1,1)^{\mathrm{T}}$	31	80	80	2.910505e − 001	2.642828e − 001
PHS48	5	2	4	$(3,3,3,3,-3)^{\mathrm{T}}$	49	140	140	3.060758e − 008	2.962501e − 001
PHS50	5	3	4	$(-3,3,3,3,3)^{\mathrm{T}}$	23	84	84	2.390072e − 009	3.139633e − 001
PHS51	5	3	4	$(-1,1,1,1,1)^{\mathrm{T}}$	13	14	14	4.687353e − 008	2.302719e − 001
PHS61	3	2	3	$(2.5,2.5,2.5)^{\mathrm{T}}$	59	59	59	−8.191909e + 001	3.401501e − 001
PHS77	5	2	4	$(1,1,1,1,1)^{\mathrm{T}}$	23	25	25	2.415051e − 001	2.393263e − 001
PHS79	5	3	4	$(-1,1,1,1,1)^{\mathrm{T}}$	44	50	50	7.877716e − 002	3.415668e − 001

**Table 2:** **The numerical results for NCM problem**

***n***	***l***	***m***	**Algorithm**	**Iter.**	**NF**	**NC**
10	5	5	Algo. A	8	15	15
			Algo. YYNY	8	-	-
			Algo. SDPIP	9	-	-
45	10	10	Algo. A	10	19	19
			Algo. YYNY	8	-	-
			Algo. SDPIP	10	-	-
105	15	15	Algo. A	10	20	20
			Algo. YYNY	10	-	-
			Algo. SDPIP	11	-	-
190	20	20	Algo. A	10	18	18
			Algo. YYNY	11	-	-
			Algo. SDPIP	12	-	-
300	25	25	Algo. A	10	25	25
			Algo. YYNY	10	-	-
			Algo. SDPIP	11	-	-
435	30	30	Algo. A	10	19	19
			Algo. YYNY	9	-	-
			Algo. SDPIP	10	-	-
595	35	35	Algo. A	11	25	25
			Algo. YYNY	11	-	-
			Algo. SDPIP	11	-	-
780	40	40	Algo. A	11	24	24
			Algo. YYNY	11	-	-
			Algo. SDPIP	11	-	-
1,225	50	50	Algo. A	12	34	34
			Algo. YYNY	−	-	-
			Algo. SDPIP	−	-	-

## Concluding remarks

We have presented a globally convergent QP-free algorithm for nonlinear SDP problems. Based on KKT conditions of nonlinear SDP problems and techniques of perturbation, we construct two SLEs skillfully. Under some linear independence condition, the SLEs have unique solution. At each iteration, the search direction is yielded by solving two SLEs with the same coefficient matrix; some penalty function is used as the merit function for line search and the penalty parameter is updated automatically in the algorithm. The preliminary numerical results show that the proposed algorithm is effective and comparable.
